# A clinical application study of a stent placement assessment

**DOI:** 10.1097/MD.0000000000031882

**Published:** 2022-11-25

**Authors:** Mingsheng Yu, Xinglu Miao, Ying Huang, Lin Ma, Long Yin, Hecheng Ren, Zengguang Wang

**Affiliations:** a Department of Neurosurgery, Tianjin Medical University General Hospital, Tianjin, China; b Department of Neurosurgery, Tianjin Huanhu Hospital, Tianjin, China; c Department of Neurosurgery, Jining No.1 People’s Hospital, Jining, Shandong Province, China.

**Keywords:** acute stroke, atherosclerosis, carotid occlusion, carotid stenting, tandem occlusion

## Abstract

Atherosclerotic acute carotid occlusion is a specific type of stroke, and controversy exists regarding the surgical strategy, that is, whether an internal carotid artery stent should be placed immediately after opening the occluded vessel. There is no objective evaluation system for this procedure. In a previous study, we summarized an evaluation decision system Emergent Carotid Artery Stent placement decision Evaluation System (ECASES) for emergency stent placement.

**Study design:**

This is a prospective, single-center, randomized controlled trial. Patients with acute ischemic stroke caused by atherosclerotic carotid artery occlusion confirmed by imaging (computed tomography/magnetic resonance angiography/digital subtraction angiography) will be randomly divided into the study and control groups, with 101 patients in each group. The study group will undergo surgery according to the ECASES system and the control group will undergo surgery according to the operator’s experience. The postoperative outcomes of the 2 groups will be compared.

**Study outcomes:**

Primary outcome: Neurological functional status (modified Rankin Scale and National Institutes of Health Stroke Scale scores) of patients 90 days postoperatively. Secondary outcomes: neurological function changes, hemorrhage events, cerebral edema, postoperative modified treatment in cerebral infarction grade, new cerebral infarction, and reocclusion of responsible vessels.

**Discussion:**

Currently, no prospective controlled data exist regarding the efficacy and safety of carotid stenting in the acute phase. Previously, we had developed an ECASES stent placement system for acute carotid artery occlusion. The present study will evaluate the efficacy and safety of ECASES in a randomized, double-blind prospective study and clarify its guiding significance in acute atherosclerotic carotid artery occlusion surgery.

## 1. Introduction

Large-vessel occlusion can cause ischemic stroke and is closely associated with poor prognosis. Intravenous thrombolysis is inefficient for recanalization of large-vessel occlusion, and intravascular intervention has become the standard treatment for acute stroke with large-vessel occlusion.^[1–3]^ Despite the rapid development of surgical techniques and materials and significant improvements in the surgical recuperation rate, many difficulties and controversies remain regarding some disease subtypes, whether in surgical techniques or strategies and are worthy of further study.

Atherosclerotic acute carotid occlusion is a specific type of large vessel, occlusive acute stroke. Its etiology is based on atherosclerotic stenosis at the beginning of the internal carotid artery and acute occlusion of the carotid artery due to hemodynamic or other causes, which is often accompanied by embolization of large intracranial vessels (tandem occlusion), resulting in acute cerebral infarction. The current surgical treatment for acute carotid occlusion in atherosclerosis is controversial at both the technical and strategic levels.^[4]^ One main controversy is whether to place a stent in the carotid artery immediately after opening the occluded vessel.^[5,6]^ The advantage of stent placement is that it guarantees patency of the vessel and reduces the risk of postoperative vessel reocclusion; however, it may increase the risk of intracranial hemorrhage owing to the application of antiplatelet agents and excessive perfusion. Without stenting, the risk of intracranial hemorrhage may be reduced; however, the risk of postoperative vessel reocclusion increases. The conclusions of the available studies are inconsistent. Some studies have shown results favoring simultaneous stent placement, but others have shown the opposite, and others have concluded that stent placement has no effect on patient prognosis.^[7,8]^

We believe that assessment and decision making should be individualized and objective for each patient and cannot be generalized. In our previous work, we summarized a decision evaluation system, Emergent Carotid Artery Stent placement decision Evaluation System (ECASES), for stent placement decisions in atherosclerotic acute carotid artery occlusion. The system is divided into 3 parts: bleeding risk score, ischemia risk score, and an individualized decision-making system that can specifically and quantitatively assess a patient’s risk of intracranial hemorrhage and ischemia and make individualized treatment recommendations.

Therefore, this study aimed to further evaluate the efficacy and safety of ECASES through a randomized, double-blind, prospective controlled study to clarify its guiding significance in acute atherosclerotic carotid artery occlusion surgery.

## 2. Methods

### 2.1. Study design

This is a prospective controlled study with a randomized, double-blind design, in which subjects will be randomized into 2 groups: the study group will undergo surgery according to the ECASES system and the control group will undergo surgery according to the operator’s experience. The postoperative results of the 2 groups will be then compared. This study was registered in the Chinese Clinical Trial Registry (Identifier: ChiCTR2200055890) and will be conducted in accordance with the Declaration of Helsinki and Good Clinical Practice guidelines.

### 2.2. Study population and preoperative evaluation

The study population will include patients with acute ischemic stroke caused by atherosclerotic carotid occlusion and confirmed by imaging (computed tomography angiography (CTA)/magnetic resonance angiography (MRA)/digital subtraction angiography (DSA)) attending Tianjin Huanhu Hospital, and 202 patients are planned to be enrolled: 101 patients in the study group and 101 patients in the control group.

1) Inclusion criteria.
  • Age 18–80 years old, regardless of sex.
  • Preoperative National Institutes of Health Stroke Scale (NIHSS) score ≥ 6.
  • An Alberta Stroke Program Early Computed Tomography Score (ASPECTS) ≥ 6 based on magnetic resonance imaging diffusion-weighted imaging (MRI-DWI) sequences.
  • The time from onset to the expected start of endovascular treatment is ≤ 24 hours, with patients between 6 and 16 hours meeting the inclusion criteria for the DAWN study and the DEFUSE 3 study, and patients between 16 and 24 hours meeting the inclusion criteria for the DAWN study.
  • Pre-stroke modified Rankin Scale (mRS) score of 0–1.
  • Life expectancy ≥ 2 years.
  • Patients and their families agree to the endovascular treatment and study protocol and sign an informed consent form.
2) Exclusion criteria.
  • Patients with combined intracranial hemorrhage confirmed by CT or MRI.
  • Patients with a combination of severe renal insufficiency (creatinine level > 264 µmol/L), respiratory and circulatory instability, severe coagulation dysfunction (international normalized ratio > 3.0, or partial thromboplastin time more than 3 times the normal value), and other contraindications to interventional procedures.
  • Patients with preexisting neurological(pre-stroke mRS score ≥ 2, stroke attack with epilepsy, combined with intracranial tumors) or psychiatric disorders could confound the results.
  • Uncontrollable hypertension is defined as a systolic blood pressure > 185 mm Hg and diastolic blood pressure > 110 mm Hg.
  • Platelet count < 50,000/µL.
  • Blood glucose < 2.78 mmol/L or > 22.20 mmol/L.
  • Patients who cannot undergo cranial MRI.
  • Patients deemed unsuitable for study participation by other investigators.
3) Exclusion and discontinuation criteria: Patients may withdraw at any stage of the study.
  • Voluntary withdrawal, where informed consent is withdrawn, and patients are free to withdraw from the study at any time without prejudice to subsequent treatment.
  • Missed patient visits.
  • Any safety reasons (adverse events) as perceived by investigators.
  • Other cases where researchers decide whether it is appropriate for patients to withdraw from the study.
4) Preoperative evaluation.
  • General information: Age, sex, time of onset, past medical history, and stroke risk factors, including blood pressure, blood glucose, blood count, coagulation series, and electrocardiogram.
  • Neurological function assessment: NIHSS and mRS scores will be used as tools for neurological function evaluation and completed by senior physicians.
  • Imaging evaluation: All patients undergo preoperative cranial CT to exclude intracranial hemorrhage and massive cerebral infarction. All patients will undergo cranial MRI to clarify the diagnosis and extent of acute cerebral infarction and will then be scored based on MRI-DWI sequences with reference to the ASPECTS scoring system. The presence of large infarcts in the basal ganglia area (large infarcts: >2/3 of the basal ganglia area) will also be assessed. All the patients will undergo MRA, CTA, and/or DSA to confirm the presence of atherosclerotic carotid occlusion. In some patients (6–24 hours after onset), CT perfusion imaging or nuclear perfusion imaging will be performed to assess the semidark bands.

### 2.3. Randomized grouping and blinding method

1. Randomized grouping method: Pass software will be used to randomize the study subjects into the study and control groups. The study subjects will be numbered, and random numbers will be generated using the pass software functions Transform-Compute (calculate new variables) - Rv.Uniform (uniformly distributed random number function). Transform-Rank Cases will be applied to generate a random number order.
2. Blinding: The study will be double-blind, blinded to the patient and investigator, and neither the patients nor investigators will know the subject grouping throughout treatment and follow-up. The randomization grouping results will be maintained by a dedicated person (stenting protocol selection decision maker), who is a nontreatment/non-follow-up participant who is aware of the patient’s grouping assignment (study or control group) and maintains patient and investigator confidentiality.

### 2.4. Interventional therapy and intraoperative evaluation

Angiography of 4 branches of the whole brain (bilateral common carotid arteries and bilateral vertebral arteries) will be performed to evaluate the primary compensation of the diseased vascular area (circle of Willis compensation: whether the posterior and/or anterior communicating arteries are open). The operation then begins with the use of an embolus protection device. After carotid artery occlusion is expanded by a percutaneous transluminal angioplasty balloon, the catheter will be guided through the internal carotid artery occlusion segment, and intracranial vascular thrombectomy and recanalization will be performed using classical thrombectomy techniques. After successful opening of the occluded vessel or thrombus retrieval, observation will be performed for 30 minutes, and common carotid angiography (lesion vessel assessment angiography) will be repeated to assess the extent, stability, and changes in stenosis at the beginning of the internal carotid artery, in parallel with DynaCT (to confirm the presence of intracranial hemorrhage or contrast spillage). Finally, the next procedural step (whether to place a carotid stent and/or apply antiplatelet agents) will be performed according to the treatment plan decision maker’s instructions.

### 2.5. Treatment plan recommendation and determination

Three surgical options are currently available. Stent placement + immediate normal application of antiplatelet drugs; Stent placement + delayed application of antiplatelet drugs; and no stent placement. “Immediate normal antiplatelet medication” refers to an intravenous tirofiban loading dose before stent placement and continuous intravenous pumping (calculated by kilogram of body weight according to the instructions) until 24 to 48 hours after surgery, followed by aspirin (100 mg), clopidogrel (75 mg), and oral quaque die. Delayed application of antiplatelet drugs means that 24 to 48 hours after surgery, when the condition is stable, as indicated by CT review, aspirin (100 mg) + clopidogrel (75 mg), and oral quaque die will be administered.

The ECASES assessor, a nonsurgical participant, will evaluate each subject (study or control group) according to the ECASES scoring rules (see below) and make ECASES treatment recommendations to the protocol decision maker; the surgeon and their assistant will evaluate and discuss each subject (study or control group) based on their experience and make treatment recommendations to the protocol decision maker. The ECASES assessor and surgical participants will be unaware of their respective treatment recommendations, and the decision maker will select a treatment plan based on subject grouping (ECASES recommendations for the study group/operator’s experience for the control group) and inform the operators of the finalized treatment plan for the next operation; however, they will not inform the operator of the source of the regimen (derived from the ECASES system or operator’s experience) and the subject subgroup.

### 2.6. ECASES

ECASES is divided into 2 risk scoring systems (bleeding risk score and ischemic risk score) and a decision recommendation system that weighs the patient’s bleeding and ischemic risks, resulting in an individualized treatment recommendation for stent placement.

1. The bleeding risk of patients is assessed based on the intraoperative DynaCT results and DWI sequence results on preoperative MRI examinations. Bleeding risk is scored as 0, 1, 2, or 3 (Table 1), with red, yellow, and blue representing high to low bleeding risk grading.

**Table 1:**
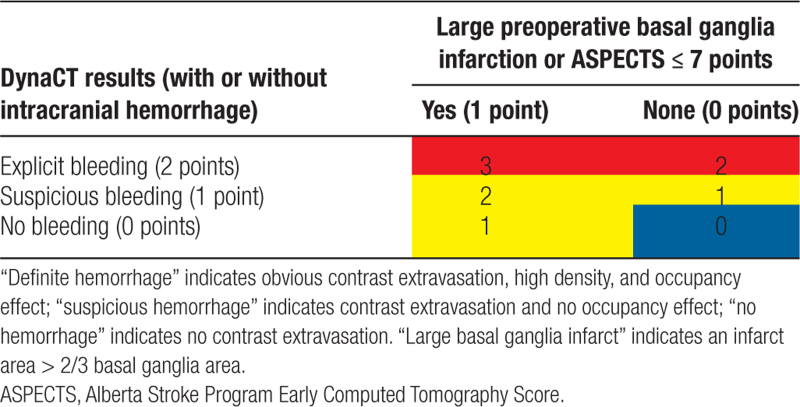
Risk score for intracranial hemorrhage.

2. The ischemic risk of patients will be assessed based on the primary vascular substitution (anterior communicating artery/posterior communicating artery) and intraoperative observation of the lesion vessels after 30 minutes of assessment angiography. Ischemic risk scores of 0, 1, 2, or 3 are assigned as shown in Table 2; red, yellow, blue, and green represent ischemic risk graded from high to low.

**Table 2:**
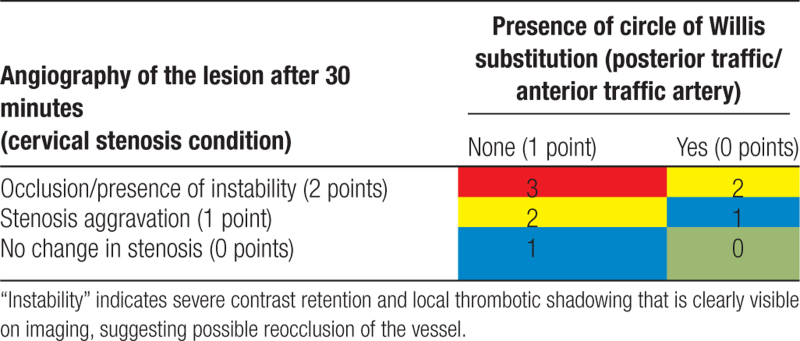
Risk score for intracranial ischemia.

3. Finally, according to the bleeding and ischemic risk assessments in Tables 1 and 2, recommendations will be made for stent placement and subsequent antiplatelet drug therapy. The color grade of bleeding risk will be first considered, then the ischemic risk will be considered, and finally, the recommendation will be given (Table 3).

**Table 3:**
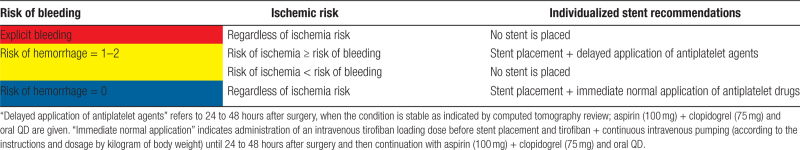
Individualized stent recommendations.

### 2.7. Postoperative evaluation and patient follow-up

1) Imaging evaluation and follow-up.
  • CT: 3 planned CT examinations will be performed on the first, third, and 7th to 14th day after surgery. Additionally, CT examinations will be performed at any time according to the disease condition to evaluate intracranial hemorrhage and cerebral edema.
  • MRI: A planned MRI examination will be performed on the 3rd to 7th days after surgery, and examinations will be performed at any time according to the disease condition to evaluate new cerebral infarction and other conditions.
  • Vascular imaging examination: 2 planned CTA, MRA, and/or DSA examinations will be performed on the 90th day, 7 to 14 days after the operation, and at any time according to the disease condition to evaluate the reocclusion of the responsible vessels.
2) Neurological function assessment and follow-up.

Patients will undergo follow-up evaluation on days 1, 7, 30, and 90 after surgery, and the NIHSS and mRS scores will be assessed.

### 2.8. Study outcomes

1. Primary outcome: Neurological functional status at 90 days postoperatively

Patient neurological functional status will be assessed by mRS and NIHSS scores.

1. Secondary outcomes.

- Changes in neurological function are classified as improved (postoperative NIHSS score decrease ≥ 4 points compared with preoperative), worsened (postoperative NIHSS score increase ≥ 4 points compared with preoperative), or stable (postoperative NIHSS score change < 4 points).
- Hemorrhage events: For the classification of bleeding events, we refer to ECASS II.^[9]^ Hemorrhagic events were classified according to the clinical and CT criteria. Hemorrhagic infarction 1 (HI1) was defined as small petechiae along the margins of the infarct, and hemorrhagic infarction 2 (HI2) as confluent petechiae within the infarcted area but no space-occupying effect.

parenchymal hemorrhage (PH1) as blood clots in 30% or less of the infarcted area with some slight space-occupying effect, and parenchymal hemorrhage (PH2) as blood clots in more than 30% of the infarcted area with a substantial space-occupying effect. Symptomatic intracranial hemorrhage was defined as blood at any site in the brain on the CT scan (as assessed by the CT reading panel, independent of the assessment by the investigator), documentation by the investigator of clinical deterioration, adverse events indicating clinical worsening (e.g., drowsiness, increase of hemiparesis), or a decrease in the NIHSS score of 4 or more points.

- Cerebral edema: According to postoperative CT results, edema is classified into 4 conditions: no edema, mild edema (focal edema up to one-third of the hemisphere), moderate edema (focal edema greater than one-third of the hemisphere), and severe edema (edema with midline shift).^[10]^ Signs of focal edema are defined as narrowing of the cerebrospinal fluid space, for example, effacement of the cortical sulci or ventricular compression.^[11]^
- Modified treatment in cerebral infarction (mTICI) grading: The mTICI grading will be assessed based on DSA of the responsible vessel at the end of the procedure to evaluate the effectiveness of surgical revascularization.
- New cerebral infarction: Postoperative MRI-DWI sequences showed a new cerebral infarction (compared with the preoperative).
- Reocclusion of the responsible vessel: A responsible artery with an immediate postoperative mTICI classification ≥ 2b and a CTA, MRI, and/or DSA examination within 90 days postoperatively, suggesting artery reocclusion, will be defined as reocclusion of the responsible vessel.

### 2.9. Data management system

All cases included in the study will be entered into the case report form by responsible evaluators within 48 hours and entered into the spreadsheet within 1 week. To clarify the person responsible, all persons entering the case report form will sign this form. A data supervisor who is not involved in the clinical study will check the data; when incorrect data are found, the original data will be searched and corrected in a timely manner. The date and person making the changes should be recorded to ensure that the data are error free.

### 2.10. Data security and monitoring board

To ensure the integrity of the clinical trial and protect the rights and health of the subjects, a data and safety monitoring committee is established for this study. Monitoring visits to clinical sites will be conducted periodically during the study to ensure that all aspects of the currently approved protocol/amendments are followed.

### 2.11. Sample size estimation

Assuming a rate of good neurological function in both the study and control groups (180 d) of not less than 51%, a noninferiority threshold Δ=–10%, a sample ratio of the study and control groups of 1:1, a unilateral test level (α = 0.025), a total certainty of 80%, and a 5% loss rate, the total sample size required is 202 patients and 101 patients in each group.

### 2.12. Statistical analysis methods

All data will be analyzed according to the intentionality analysis and will follow the protocol analysis statistics. Statistical descriptions will be used to compare the baseline information. The mean ± standard deviation or median (interquartile range) will be used to express the measurement data, and rates or percentages will be used for count data. For measurement data, a *t* test or Mann–Whitney *U* test will be used to detect differences between the groups. The Chi-squared test will be used to analyze the count data.

All data will be analyzed using SPSS 23.0 (IBM Corporation, Armonk, NY) software, and *P* < .05 (2-sided) will be considered a statistically significant difference.

### 2.13. Technology line

**Figure 1.**
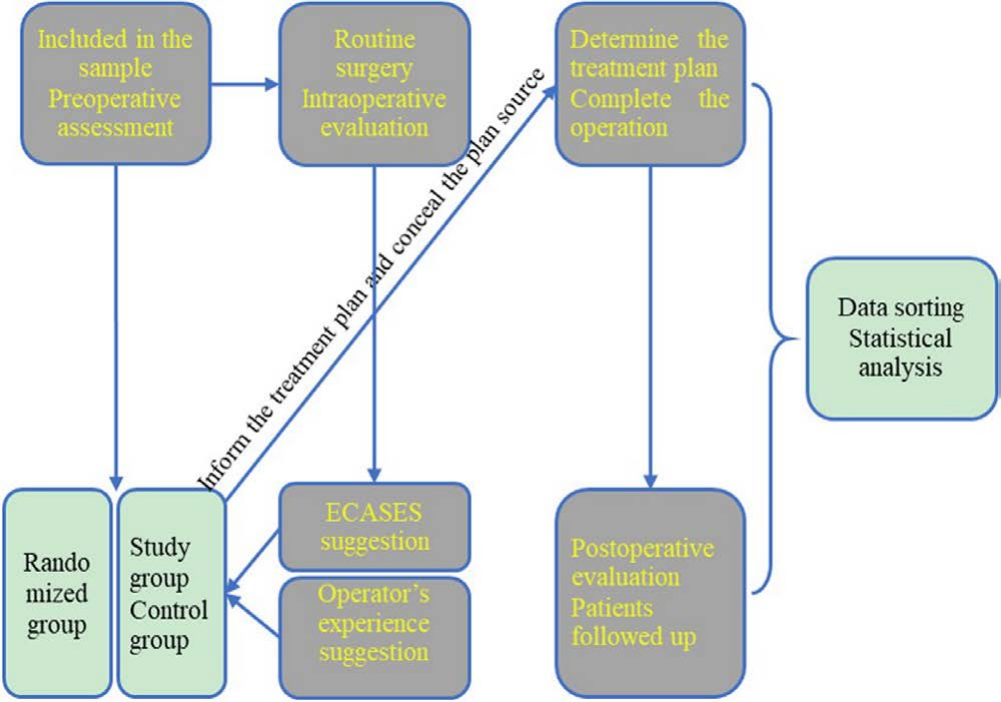
Clinical intervention technology roadmap.

The clinical intervention technology roadmap is shown in Fig. 1.

## 3. Discussion

Acute carotid stenting procedures associated with arterial thrombectomy have been performed by many neurointerventionists. The TITAN study, a retrospective pooled analysis of data from acute ischemic stroke patients with tandem lesions in the anterior circulation from 18 international comprehensive stroke centers (including more than 450 patients), showed that acute stenting of an extracranial internal carotid artery lesion was associated with improved reperfusion (mTICI 2b-3 and mTICI 3)^[12,13]^ without an excess risk of intracranial hemorrhage or mortality,^[14]^ as previously reported in 2 meta-analyses of case series.^[15,16]^ However, prospective controlled data are lacking regarding the efficacy and safety of carotid stenting in the acute phase of stroke reperfusion, particularly in the context of antiplatelet therapy.

According to research studies and work experience, intracranial hemorrhage is related to the preoperative infarct size, infarct site (basal ganglia area), contrast medium extravasation, or bleeding detected by intraoperative DynaCT after vascular recanalization in acute stroke with large-vessel occlusion.^[17,18]^ Postoperative recovery of neurological function (improvement of cerebral ischemia) is related to postoperative vascular patency and collateral circulation compensation.^[19]^ Accordingly, the ECASES system proposes a bleeding risk score and an ischemic risk score based on the above factors and then suggests an individualized stenting decision by weighing bleeding and ischemic risks with a sufficient theoretical basis. This study uses a randomized, double-blind design in which, for each subject, 2 types of stenting options from the ECASES system and expert experience are provided by investigators, and the stenting option is selected by a dedicated person (nonsurgical participant) according to the subject group. This person then informs the surgical staff, but the source of the option is not revealed, which minimizes confounding factors and bias and results in a reasonable and reliable study design. Additionally, ECASES uses a scale format that can realize specific and quantitative evaluations, and is accurate, fast, and efficient.

## Author contributions

**Conceptualization:** Hecheng Ren, Zengguang Wang.

**Formal analysis:** Lin Ma.

**Supervision:** Ying Huang, Long Yin.

**Writing—original draft:** Mingsheng Yu, Xinglu Miao.

**Writing—review and editing:** Hecheng Ren, Zengguang Wang.
